# Probe Majorana zero modes through their spins

**DOI:** 10.1093/nsr/nwy152

**Published:** 2018-12-17

**Authors:** Yi Zhou

**Affiliations:** Department of Physics, Zhejiang University, China

## MAJORANA PARTICLE VS. MAJORANA MODE

A Majorana particle is a fermion that is identical to its own antiparticle. Majorana fermions are named after Ettore Majorana, who predicted this type of particle in 1937 [1]. He suggested that charge-neutral spin-1/2 particles can be described by a real wave equation as opposed to the Dirac equation, which is a complex wave equation, and proposed that neutrinos are particles of such a type. Since the particle–antiparticle transformation will change the sign of the charge, a Majorana particle must be charge neutral. To the best of our knowledge, all the standard model fermions in particle physics are known as Dirac fermions, except the neutrino. The latter may be either a Dirac or a Majorana fermion, and the issue has not yet been settled [2].

Majorana zero mode (MZM), also referred to as the zero-energy Majorana bound state, is a property of existing particles than a new particle, in the sense that a particle should have a well defined energy–momentum dispersion relation. Indeed, the word ‘mode’ indicates a solution to the wave equation in a quantum system, which could be either Schrödinger or Dirac-like. Majorana fermions obey fermionic statistics, while a particle (to be precise, a particle-like object) associated with the Majorana mode obeys non-Abelian statistics [3]. Interchanging two such particles will not only change the phase of the wave function, but also change the internal state of the modes.

## MAJORANA ZERO MODES IN SOLID-STATE PHYSICS

Over the past two decades, Majorana modes in the solid state have received more and more attention. Note that holes in a solid can be viewed as antiparticles of valence electrons. Thus, an equal-weight superposition of an electron and a hole allows us to construct a Majorana mode in a condensed-matter system. In condensed-matter physics, such an electron–hole superposition is known as a Bogoliubov quasiparticle in a superconductor. However, Bogoliubov quasiparticles are not exactly their own antiparticles, except in accidental situations. This is the reason why Majorana modes were not realized in superconductors for more than 40 years although Bogoliubov quasiparticles were well established.

**Figure 1.**
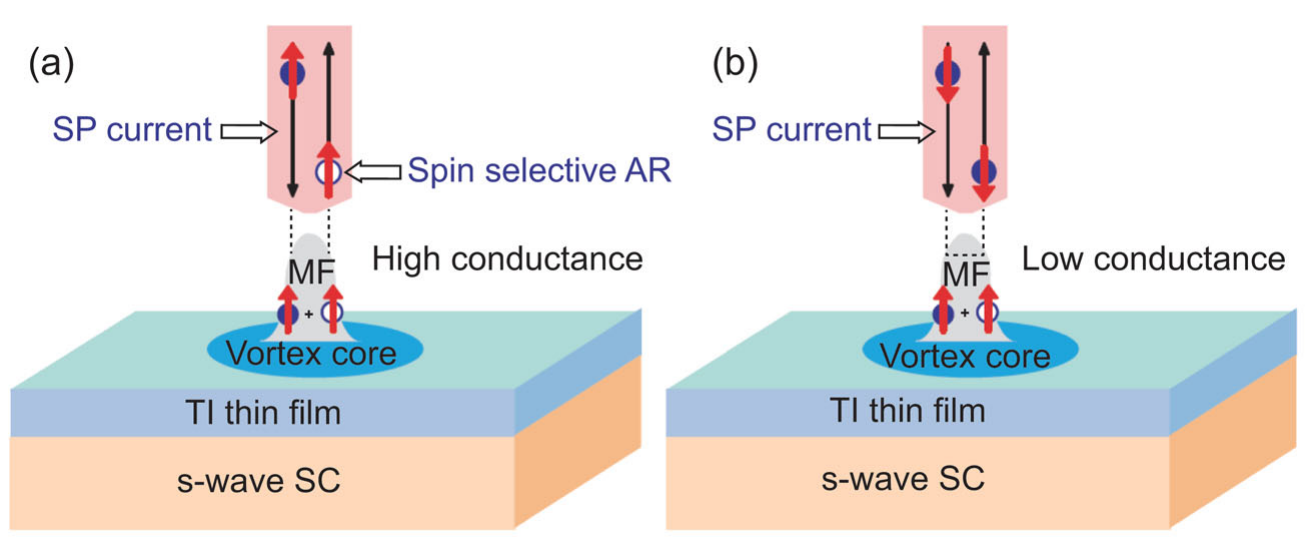
Illustration of spin-selective Andreev reflection in spin-polarized STM/scanning tunneling spectroscopy on a vortex center in a 2D topological superconductor, which is an effective chiral p-wave superconductor induced by the proximity effect at the surface of a 3D topological insulator. (a) An incoming spin-up electron of zero energy is reflected as an outgoing spin-up hole induced by the Majorana zero mode with spin-up, which gives out a higher tunneling conductance. (b) An incoming spin-down electron of zero energy is reflected directly because of the mismatch of the spins of the electron and the Majorana zero mode, which results in a lower tunneling conductance. (Taken from [19].)

The situation changed about 20 years ago, when people found that a Majorana mode may emerge as a very robust zero-energy bound state in the vortex core of a ${p_x} + {\rm{i}}{p_y}$ superconductor. Indeed, such a vortex could be either a single-quantum (${\rm{hc}}/2{\rm{e}}$) vortex in a spinless superconductor [4] or, equivalently, a half-quantum (${\rm{hc}}/4{\rm{e}}$) vortex in a spinful superconductor [5,6]. As mentioned, such vortices carrying MZMs obey non-Abelian statistics and were proposed as topological qubits in quantum computation [7]. It was proposed by Fu and Kane that an effective chiral p-wave superconductor can be induced by the proximity effect at the surface of a 3D strong topological insulator [8]. Such a 2D topological superconductor has been realized in the Bi_2_Te_3_/NbSe_2_ heterostructure and a zero-energy peak has been observed in scanning tunneling microscopy (STM) tunneling spectra in the vortex core, suggesting the existence of MZMs [9,10]. It is worth noting that there are other solid-state candidates hosting MZMs besides chiral p-wave superconductors, including the ${\rm{\nu \ }} = {\rm{\ }}5/2$ fractional quantum Hall system [3,4], proximity-induced superconductors in nanowires [11,12], spin–orbit Rashba-coupled semiconducting nanowires [13–15], and ferromagnetic atomic chains in proximity to superconductors [16,17].

## PROBING MAJORANA ZERO MODES THROUGH SPIN DEGREES OF FREEDOM

In solid states, MZMs emerge from electrons, which carry not only charges but also spins. It was pointed out by He *et al*. that the spin degrees of freedom of MZMs can be revealed by so-called MZM-induced spin-selective Andreev reflection (SSAR) [18]. They considered a device in which a metal lead couples to a 1D topological superconductor through its MZM end state, which consists of an electron component with spin along a certain direction $\vec{n}$. An electron injected from the metal with spin polarization $\vec{n}$ will be reflected as a hole with the same spin, while an electron with opposition spin polarization $- \vec{n}$ will not participate in such Andreev reflection. This is because only spin-$\vec{n}$ polarized electrons can couple to the MZM. This is in sharp contrast to ordinary Andreev reflection processes, where any injected electron will be reflected as a hole with opposite spin. Moreover, the resonance through the MZM will give rise to a $2{{\rm{e}}^2}/{\rm{h}}$ quantized zero-energy peak in the tunneling differential conductance ${\rm{d}}I/{\rm{d}}V$.

Such MZM-induced SSAR was recently observed by Sun *et al*. in experiments [19], slightly different from the original theoretical proposal, where a metal lead couples to a nanowire system through its MZM end state; experimentalists applied spin-polarized STM to measure SSAR in a 2D topological superconductor, say, the Bi_2_Te_3_/NbSe_2_ heterostructure, as illustrated in Fig. 1. The spin polarization is realized by ferromagnetic Fe-coated W tips. The observation of a zero-bias peak (ZBP) in the tunneling conductance indicates the existence of MZM. It was reported that the intensity of the ZBP at the vortex center is substantially (14%) higher when the tip polarization and the external magnetic field are parallel rather than antiparallel to each other. Note that there always exist normal states in the vortex core center where the superfluid density vanishes, which unavoidably contribute to the ZBP. The experimental observation is in good agreement with a model calculation, in which the normal conductance is included in the total conductance in addition to the Andreev reflection [20].

## FURTHER ISSUES

Although the spin-dependent tunneling effect in a 2D topological superconductor provides direct evidence of MZM and reveals its magnetic property, there still remain some other issues to be addressed. (i) How can the expected $2{{\rm{e}}^2}/{\rm{h}}$ quantized ZBP in the tunneling conductance in 2D topological superconductors compared to that in the 1D nanowire be observed [21]? (ii) How can braiding and fusion of MZMs be realized and how are they to be probed? (iii) It is worth noting that braiding or fusion of MZMs will not be able to do universal quantum computation. How to combine the braiding operation with other quantum manipulations with topological qubits made of MZMS to realize universal quantum computation is an open question.
